# Supplementary material for: Seroprevalence of antibodies against SARS-Cov-2 in the high impacted sub-district in Jakarta, Indonesia
Source: PLoS One. 2021 Dec 23;16(12):e0261931. doi: 10.1371/journal.pone.0261931 (PMC8699601; doi:10.1371/journal.pone.0261931)
Supplement: S1 Appendix — (DOCX) [file pone.0261931.s001.docx]

**S1 Appendix. Survey Questionnaire in Indonesian and English**

| **KUESIONER RISET SEROPREVALENSI** | | |
| --- | --- | --- |
| Pernyataan | Center for Indonesia’s Strategic Development Initiatives (CISDI) merupakan organisasi masyarakat sipil yang bekerja di sektor pembangunan kesehatan. Melalui program Pencerah Nusantara, CISDI berupaya membantu pemerintah untuk melacak kasus, menekan angka kesakitan dan kematian, serta meminimalisir dampak dari pandemik COVID-19. Sebagai bagian dari mengukur dampak program Pencerah Nusantara, CISDI melakukan survei seroprevalensi menggunakan *rapid test* antibody di Tanjung Priok, Jakarta Utara sebagai wilayah intervensi. Survei seroprevalensi bertujuan untuk melihat seberapa besar sebuah populasi terinfeksi COVID-19 berdasarkan antibodi yang terbentuk.   Data yang Anda berikan bersifat rahasia. Pengisian formulir ini menjadi persyaratan pelaksanaan skrining masyarakat yang dilakukan CISDI. Hasil pemeriksaan COVID-19 dan data identitas yang bersifat pribadi yang diisikan dalam kuesioner ini tidak akan disebarluaskan ke pihak umum dan hanya diperuntukkan untuk evaluasi program, pembuatan kajian dan rekomendasi kepada pemerintah, dan publikasi ilmiah sesuai kebutuhan CISDI.  Pengisian formulir ini akan memerlukan waktu sekitar 5-10 menit. Terima kasih atas kesediaannya mengisi formulir ini. | |
| Lembar Persetujuan | Saya setuju untuk: (1) mengisi formulir berikut dan memberikan informasi yang sebenar-benarnya; (2) membolehkan penggunaan data hasil pengisian formulir berikut hanya untuk keperluan evaluasi dan pengembangan program, pembuatan kajian dan rekomendasi kepada pemerintah, dan publikasi ilmiah dengan selalu tetap menjaga kerahasiaan identitas.  - Ya, saya setuju | |
| **No** | **Pertanyaan** | **Pilihan Jawaban** |
| **BLOK 1** | **Data Enumerator** |  |
| 1.1 | Nama | (isian) |
| 1.2 | Tanggal pengambilan data | (DD/MM/YY) |
| **BLOK 2** | **Karakteristik Demografi Individu** |  |
| 2.1 | Nama lengkap sesuai KTP (Hanya huruf kecil, tanpa gelar, bukan nama panggilan) | (isian) |
| 2.2 | Kode unik individu | (isian) |
| 2.3 | Status responden | 1. Responden undangan 2. Responden *on the spot* |
| 2.4 | Nama Kepala Rumah Tangga (KRT) | (isian) |
| 2.5 | Kelurahan | (isian) |
| 2.6 | RW | (isian) |
| 2.7 | RT | (isian) |
| 2.8 | Alamat lengkap | (isian) |
| 2.9 | Nomor HP/*Whatsapp* | (isian) |
| 2.10 | Tanggal lahir | (DD/MM/YY) |
| 2.11 | Jenis kelamin | 1. Laki-laki 2. Perempuan |
| 2.12 | Status perkawinan | 1. Belum kawin 2. Kawin 3. Cerai hidup 4. Cerai mati |
| 2.13 | Apa jenjang pendidikan tertinggi yang pernah/sedang diikuti? | 0. Belum/tidak pernah sekolah 🡪 *Lanjut ke pertanyaan 2.15* 1. Paket A / SDLB / SD /MI 2. Paket B / SMPLB / SMP / MTs 3. Paket C / SMLB / SMA / MA / SMK / MAK 4. D1/D2 5. D3 6. D4 / S1 7. Profesi 8. S2 9. S3 |
| 2.14 | Apa tingkat/kelas tertinggi yang pernah atau sedang diduduki?  Kode untuk pertanyaan 2.14:   1. 1 2. 2 3. 3 4. 4 5. 5 6. 6 atau masih kuliah S2 7. Masih kuliah S3 8. Tamat dan lulus | (isian) |
| 2.15 | Apa kegiatan utama Anda selama dua minggu terakhir? | 1. Bekerja 2. Ibu/bapak rumah tangga 🡪 *Lanjut ke pertanyaan A01* 3. Sekolah/kuliah 🡪 *Lanjut ke pertanyaan A01* 4. Pengangguran/sedang mencari kerja 🡪 *Lanjut ke pertanyaan A01* 5. lainnya 🡪 *Lanjut ke pertanyaan A01* |
| 2.16 | Apa jenis pekerjaan Anda? | 1. Berusaha (usaha sendiri) 2. Berusaha dibantu buruh tidak tetap/ buruh tidak dibayar 3. Berusaha dibantu buruh tetap/ buruh dibayar 4. Buruh/karyawan/pegawai negeri 5. Buruh/karyawan/pegawai swasta 6. Pekerja bebas 7. Pekerja keluarga/tidak dibayar |
| 2.17 | Dalam sebulan terakhir, bagaimana sistem pekerjaan Anda di masa COVID-19? | 1. Boleh bekerja dari rumah 2. Piket atau bergantian ke tempat kerja 3. Harus tetap pergi ke tempat kerja atau melakukan pekerjaan seperti biasa (misal. tetap berdagang keliling atau bertemu pelanggan) 4. Dirumahkan |
| **BLOK A** | **Riwayat Tes COVID-19** |  |
| A01 | Apakah Anda pernah melakukan tes *swab* atau tes PCR? | 1. Pernah 2. Tidak 🡪 *Lanjut ke pertanyaan A04* |
| A02 | Kapan Tindakan/tes PCR tersebut dilakukan? | 1. Kurang dari 1 minggu yang lalu 2. 1-2 Minggu yang lalu 3. 3-4 Minggu yang lalu 4. 1-2 Bulan yang lalu 5. Lebih dari 2 bulan yang lalu |
| A03 | Bagaimana hasil tes *swab* atau tes PCR tersebut? | 1. Positif 2. Negatif 3. Belum keluar 4. Tidak tahu  5. Tidak mau menjawab |
| A04 | Apakah Anda pernah melakukan *rapid test* antibodi atau serologi dengan pengambilan sampel darah? | 1. Pernah 2. Tidak 🡪 *Lanjut ke pertanyaan B01* |
| A05 | Kapan *rapid test* antibody/serologi tersebut dilakukan? | 1. Kurang dari 1 minggu yang lalu 2. 1-2 Minggu yang lalu 3. 3-4 Minggu yang lalu 4. 1-2 Bulan yang lalu 5. Lebih dari 2 bulan yang lalu |
| A06 | Bagaimana hasil *rapid test* terakhir? | 1. Reaktif 2. Non-reaktif 3. Belum keluar 4. Tidak tahu  5. Tidak mau menjawab |
| **BLOK B** | **Gejala 14 Hari Terakhir** |  |
| B01 | Dalam 14 hari terakhir, apakah Anda mengalami gejala sebagai berikut?  **(JAWABAN BOLEH LEBIH DARI SATU)** | *-* Kehilangan kemampuan mencium bau atau mengecap rasa - Demam  - Nyeri otot - Menggigil atau Meriang - Batuk - Hidung berair atau hidung mampet (rhinorrhoea) - Sakit kepala - Keringat di malam hari - Diare - Sakit tenggorokan - Sesak napas - Sakit dada - Jantung berdebar - Berdahak (sputum) - Pusing berputar (dizziness) - Mual - Muntah - Nyeri perut - Mata berair/merah/bengkak - Ruam kulit/kulit kemerahan - Kehilangan nafsu makan - Kesemutan dan/atau mati rasa - Kebingungan mental (delirium) - Kejang (bukan penyakit bawaan) - Perasaan tidak enak badan (malaise) - Kelelahan berlebih (fatigue) - Berkurangnya kemampuan pendengaran yang mendadak - Mata merah - Tidak mengalami gejala 🡪 *Lanjut ke pertanyaan C01* |
| B02 | Kapan gejala paling awal tersebut muncul? | - 0 hari yang lalu (mulai hari ini) - 1 hari yang lalu - 2 hari yang lalu - 3 hari yang lalu - 4 hari yang lalu - 5 hari yang lalu - 6 hari yang lalu - 7 hari yang lalu - 8 hari yang lalu - 9 hari yang lalu - 10 hari yang lalu - 11 hari yang lalu - 12 hari yang lalu - 13 hari yang lalu |
| **BLOK C** | **Riwayat Penyakit** |  |
| C01 | Apakah saat ini Anda sedang hamil?  **(Jika 2.11=2)** | 1. Ya 2. Tidak |
| C02 | Apakah saat ini Anda memiliki kondisi/penyakit berikut atas hasil diagnosis dokter?  **(JAWABAN BOLEH PILIH LEBIH DARI SATU)** | - Penyakit paru - Stroke atau penyakit otak dan pembuluh darah otak - Gagal ginjal - Jantung koroner atau penyakit jantung lainnya - Diabetes mellitus/kencing manis/penyakit gula - Hipertensi/darah tinggi - Kanker - Gangguan liver - Gangguan sistem imun - Epilepsi/kejang berkelanjutan - Gangguan endokrin lainnya - HIV  - Lainnya, sebutkan ……………… - Tidak ada kondisi penyerta/penyakit |
| C03 | Dalam 1 bulan terakhir, apakah Anda merokok tembakau? | 1. Ya, setiap hari 2. Ya, tidak setiap hari 3. Tidak 🡪 *Lanjut ke pertanyaan C05* |
| C04 | Dalam 1 bulan terakhir terakhir, berapa batang rokok rata-rata per minggu yang Anda hisap? | (isian) |
| C05 | Apakah dulu, sebelum sebulan terakhir, pernah merokok tembakau? | 1. Ya, pernah merokok setiap hari 2. Ya, pernah merokok tidak setiap hari 3. Tidak pernah |
| C06 | Sebelum memutuskan untuk berhenti merokok, berapa batang rokok rata-rata per minggu yang Anda hisap?  **(Jika C03 = 3 DAN C05 = 1 atau 2)** | (isian) |
| C07 | Dalam 14 hari terakhir, apakah Anda pernah mengalami kontak dengan orang yang mengalami demam, batuk/pilek, atau sesak nafas? | 1. Ya, pernah 2. Tidak 🡪 *Lanjut ke pertanyaan D01* |
| C08 | Jika Ya, sebutkan siapa dan apa hubungannya  **(JAWABAN BOLEH PILIH LEBIH DARI SATU)** | 1. Suami/istri 2. Anak 3. Orang tua 4. Orang lain yang tinggal serumah 5. Tetangga 6. Rekan kerja 7. Lainnya, sebutkan ………………….. |
| **BLOK D.** | **Perilaku Kebersihan, Penggunaan Alat Pelindung Diri (APD), dan Jaga Jarak** |  |
| Perilaku Cuci Tangan | | |
| D01 | Dalam 1 bulan terakhir, apakah anda menerapkan cuci tangan **menggunakan sabun atau *hand sanitizer***? | 1. Ya, selalu 2. Ya, sering 3. Ya, kadang-kadang 4. Tidak pernah (hanya menggunakan air) 🡪 *Lanjut ke pertanyaan D13* |
| D02-D10 | Dalam 1 bulan terakhir, apakah Anda mencuci tangan menggunakan sabun atau hand sanitizer pada saat: | |
| D02 | setelah berada di tempat umum atau menyentuh barang di luar rumah? | 1. Ya, selalu 2. Ya, seringkali 3. Ya, kadang-kadang 4. Tidak 5. Tidak pernah berada di tempat umum |
| D03 | sebelum makan atau menyiapkan makanan? | 1. Ya, selalu 2. Ya, seringkali 3. Ya, kadang-kadang 4. Tidak |
| D04 | setelah menggunakan toilet? | 1. Ya, selalu 2. Ya, seringkali 3. Ya, kadang-kadang 4. Tidak |
| D05 | setelah menyentuh hewan? | 1. Ya, selalu 2. Ya, seringkali 3. Ya, kadang-kadang 4. Tidak 5. Tidak pernah menyentuh hewan |
| D06 | setelah menyentuh atau membuang sampah? | 1. Ya, selalu 2. Ya, seringkali 3. Ya, kadang-kadang 4. Tidak 5. Tidak pernah membuang sampah |
| D07 | sebelum memakai masker? | 1. Ya, selalu 2. Ya, seringkali 3. Ya, kadang-kadang 4. Tidak 5. Tidak pernah pakai masker 🡪 *lanjut ke pertanyaan D09* |
| D08 | setelah melepaskan masker? | 1. Ya, selalu 2. Ya, seringkali 3. Ya, kadang-kadang 4. Tidak |
| D09 | setelah menerima barang dari luar rumah (paket kurir, barang dari tetangga, belanjaan, uang kembalian, dll)? | 1. Ya, selalu 2. Ya, seringkali 3. Ya, kadang-kadang 4. Tidak 5. Tidak pernah menerima barang dari luar rumah |
| D10 | setelah bersalaman dengan orang lain? | 1. Ya, selalu 2. Ya, seringkali 3. Ya, kadang-kadang 4. Tidak 5. Tidak pernah bersalaman dengan orang lain |
| D11 | Dalam satu bulan terakhir, apakah Anda selalu membawa *hand sanitize*r jika keluar rumah? | 1. Ya, selalu  2. Ya, seringkali 3. Ya, kadang-kadang 4. Tidak/tidak punya 🡪 *Lanjut ke pertanyaan D13* |
| D12 | Apakah Anda dapat memperlihatkan *hand sanitizer* yang selalu Anda bawa?  **(ENUMERATOR MELAKUKAN OBSERVASI)** | 1. Ya, responden dapat memperlihatkan *hand sanitizer* 2. Responden tidak dapat memperlihatkan *hand sanitizer* |
| Perilaku Penggunaan Masker | | |
| D13 | Dalam 1 bulan terakhir, apakah Anda menggunakan masker ketika bertemu orang lain yang tidak serumah?  (contoh: saat berbincang dengan tetangga sekitar rumah) | 1. Ya, selalu 2 Ya, seringkali 3. Ya, kadang-kadang 4. Tidak 🡪 *Lanjut ke pertanyaan D19* |
| D14 | Dalam satu bulan terakhir, apa jenis masker yang paling sering Anda gunakan saat bertemu dengan orang lain yang tidak serumah? | 1. Masker N95 🡪 *Lanjut ke pertanyaan D16* 2. Masker bedah/masker satu kali pakai lainnya 🡪 *Lanjut ke pertanyaan D16* 3. Masker kain |
| D15 | Dalam satu bulan terakhir, bagaimana jenis masker kain yang paling sering Anda gunakan? | 1. Satu lapis 2. Dua lapis 3. Tiga lapis 4. Tidak tahu |
| D16 | Dalam 1 bulan terakhir, berapa kali Anda biasanya mengganti masker ketika berkegiatan di luar rumah? | 1. Lebih dari 1 kali sehari 2. 1 kali sehari 3. 1 kali dalam 2 hari atau lebih 4. Tidak diganti dalam 1 bulan terakhir |
| D17 | Dalam satu bulan terakhir, apakah Anda menurunkan/melepas masker ketika berbicara dengan orang lain yang tidak tinggal serumah? | 1. Ya, selalu 2. Ya, seringkali 3. Ya, kadang-kadang 4. Tidak pernah |
| D18 | Dalam satu bulan terakhir, apakah Anda menurunkan/melepas masker ketika bertemu/berkumpul dengan orang lain yang Anda kenal atau Anda hormati (selain orang yang tinggal serumah)? | 1. Ya, selalu 2. Ya, seringkali 3. Ya, kadang-kadang 4. Tidak pernah |
| D19 | Responden wajib menggunakan masker saat hadir ke tempat tes serologi Observasi bentuk penggunaan masker responden!  **(ENUMERATOR MELAKUKAN OBSERVASI)** | 1. Masker digunakan di bawah hidung (menutupi mulut dan dagu) 2. Masker turun ke dagu / leher 3. Masker menutupi hidung dan mulut, tetapi tidak menutupi dagu 4. Masker kendur hingga sisi kiri dan kanan terbuka 5. Masker pas di wajah (tidak kendur) DAN menutupi hidung, mulut, dan dagu 6. Tidak memakai masker |
| Perilaku Jaga Jarak fisik | | |
| D20 | Dalam 1 bulan terakhir, apakah Anda berjabat tangan atau cium tangan jika bertemu dengan orang lain yang tidak tinggal serumah? | 1. Ya, selalu 2. Ya, seringkali 3. Ya, kadang-kadang 4. Tidak |
| D21 | Dalam 1 bulan terakhir, apakah Anda menjaga jarak minimal 1 meter dengan orang lain yang tidak tinggal serumah? | 1. Ya, selalu 2. Ya, seringkali 3. Ya, kadang-kadang 4. Tidak |
| D22 | Dalam satu bulan terakhir, apakah Anda tetap menjaga jarak fisik minimal 1 meter dengan orang lain jika telah menggunakan masker?  **(Jika D13=1 atau 2 atau 3)** | 1. Ya, selalu 2. Ya, seringkali 3. Ya, kadang-kadang 4. Tidak pernah |
| D23 | Dalam 1 bulan terakhir, apakah Anda pernah berkumpul bersama lebih dari 2 orang yang tidak tinggal serumah  (misal: makan bersama, syukuran, pengajian, reuni, arisan, dll.)? | 1. Ya, sangat sering (lebih dari 6 kali dalam seminggu) 2. Ya, sering (4-6 kali dalam seminggu) 3. Ya, kadang-kadang (1-3 kali dalam seminggu) 4. Ya, jarang (1-3 kali dalam sebulan) 5. Tidak pernah |
| D24 | Dalam 1 bulan terakhir, apakah Anda pernah bepergian dalam rangka rekreasi/jalan-jalan ke tempat hiburan (pusat pembelanjaan, dll), makan/minum di restoran/kafe, atau tempat wisata (kebun binatang, taman, *car free day*, dll)? | 1. Ya, sangat sering (lebih dari 6 kali dalam seminggu) 2. Ya, sering (4-6 kali dalam seminggu) 3. Ya, kadang-kadang (1-3 kali dalam seminggu) 4. Ya, jarang (1-3 kali dalam sebulan) 5. Tidak pernah |
| D25 | Dalam 1 bulan terakhir, seberapa sering Anda melakukan ibadah di tempat ibadah umum (masjid, gereja, pura, dll.)? | 1. Sangat sering (lebih dari 6 kali dalam seminggu) 2. Sering (4-6 kali dalam seminggu) 3. Kadang-kadang (1-3 kali dalam seminggu) 4. Jarang (1-3 kali dalam sebulan) 5. Tidak pernah |
| D26 | Dalam 1 bulan terakhir, apakah Anda pernah menumpang transportasi umum masal (Contohnya angkutan kota, MRT, bus, atau KRL)? | 1. Ya, sangat sering (lebih dari 6 kali dalam seminggu) 2. Ya, sering (4-6 kali dalam seminggu) 3. Ya, kadang-kadang (1-3 kali dalam seminggu) 4. Ya, jarang (1-3 kali dalam sebulan) 5. Tidak pernah |
| D27 | Dalam 1 bulan terakhir, seberapa sering Anda berbelanja kebutuhan pokok di pasar atau *supermarket*? | 1. Sangat sering (lebih dari 6 kali dalam seminggu) 2. Sering (4-6 kali dalam seminggu) 3. Kadang-kadang (1-3 kali dalam seminggu) 4. Jarang (1-3 kali dalam sebulan) 5. Tidak pernah |
| D28 | Dalam 1 bulan terakhir, seberapa sering Anda berkunjung ke fasilitas kesehatan (klinik, puskesmas, rumah sakit, praktik dokter/bidan)? | 1. Sangat sering (lebih dari 6 kali dalam seminggu) 2. Sering (4-6 kali dalam seminggu) 3. Kadang-kadang (1-3 kali dalam seminggu) 4. Jarang (1-3 kali dalam sebulan) 5. Tidak pernah |

| **SEROPREVALENCE RESEARCH QUESTIONNAIRE** | | |
| --- | --- | --- |
| Statement | The Center for Indonesia's Strategic Development Initiatives (CISDI) is a civil society organization working in the health development sector. Through the Pencerah Nusantara program, CISDI seeks to help the government to track cases, reduce morbidity and mortality rates, and minimize the impact of the COVID-19 pandemic. As part of measuring the impact of the Enlightenment Nusantara program, CISDI is conducting a seroprevalence survey using a rapid antibody test in Tanjung Priok, North Jakarta as the area of intervention. The seroprevalence survey aims to examine the proportion of the population infected with COVID-19 based on the antibodies formed.  The data you provide is confidential. Filling out this form is a requirement for the implementation of community screening conducted by CISDI. The results of the COVID-19 examination and personal identity data provided in this questionnaire will not be disclosed to the public and are only intended for program evaluation, writing up studies and recommendations to the government, and scientific publications in accordance with the needs of CISDI.  This survey will take approximately 5-10 minutes.  Thank you for your willingness to participate in this survey. | |
| Consent Sheet | I agree to:  (1) fill out the following form and provide truthful information;  (2) allow the use of data resulting from filling out the following form only for the purposes of program evaluation and development, writing up studies and recommendations to the government, and scientific publications while always maintaining the confidentiality of identities.  - Yes, I agree | |
| **Number** | **Question** | **Answer Options** |
| **BLOCK 1** | **Data Enumerator** |  |
| 1.1 | Name | (open-ended) |
| 1.2 | Date of data collection | (DD/MM/YY) |
| **BLOCK 2** | **Individual Demographic Characteristics** |  |
| 2.1 | Full name as it appears on ID card  (Lowercase only, no titles, not nicknames) | (open-ended) |
| 2.2 | Individual unique code | (open-ended) |
| 2.3 | Respondent status | 1. By invitation 2. On the spot |
| 2.4 | Name of Head of Household | (open-ended) |
| 2.5 | *Kelurahan* / Urban village | (open-ended) |
| 2.6 | *Rukun Warga* / Hamlet | (open-ended) |
| 2.7 | *Rukun Tetangga* / Neighbourhood | (open-ended) |
| 2.8 | Full address | (open-ended) |
| 2.9 | Mobile number/*Whatsapp* | (open-ended) |
| 2.10 | Date of birth | (DD/MM/YY) |
| 2.11 | Gender | 1. Male 2. Female |
| 2.12 | Marriage status | 1. Single 2. Married 3. Divorced 4. Widowed |
| 2.13 | What is the highest level of education that you attended/are currently attending? | 1. Haven't/never been to school 🡪 *Continue to question 2.15* 2. Elementary School Equivalency Test A / Special Needs Elementary School / Elementary School / Islamic Elementary School 3. Junior High School Equivalency Test B / Special Needs Junior High School / Junior High School / Islamic Junior High School 4. Senior High School Equivalency Test C / Special Needs Senior High School/ Senior High School / Islamic Senior High School / Vocational School / Islamic Vocational School 5. Associate Degree 1 / Associate Degree 2 6. Associate Degree 3 7. Associate Degree 4 / Bachelor 8. Professional schools 9. Master’s degree 10. Doctoral degree |
| 2.14 | What is the highest level/grade you attended or are currently attending?  Code for question 2.14:   1. 1 2. 2 3. 3 4. 4 5. 5 6. 6 or still studying a Master’s degree 7. Still undertaking a Doctoral degree 8. Graduated | (open-ended) |
| 2.15 | What has been your main activity over the last two weeks? | 1. Working 2. Housewife/househusband 🡪 *Continue to question A01* 3. School/college/university 🡪 *Continue to question A01* 4. Unemployed/looking for work 🡪 *Continue to question A01* 5. Others 🡪 *Continue to question A01* |
| 2.16 | What is your type of work? | 1. Self-employed (solopreneur) 2. Self-employed assisted by non-permanent workers/unpaid workers 3. Self-employed assisted by permanent workers/paid workers 4. Worker/employee/civil servant 5. Worker/employee/private employee 6. Casual worker/freelancer 7. Family/unpaid worker |
| 2.17 | In the past month, what was your work arrangement during COVID-19? | 1. Allowed to work from home 2. Alternating or taking turns working from the office 3. Must continue to go to work or do business as usual (eg continue to sell products by traveling around or meeting customers) 4. Dismissed |
| **BLOCK A** | **COVID-19 Test History** |  |
| A01 | Have you ever had a swab test or a PCR test? | 1. Yes, I have. 2. No, I haven’t 🡪 *Continue to question A04* |
| A02 | When was the swab test/PCR test done? | 1. Less than 1 week ago 2. 1-2 weeks ago 3. 3-4 weeks ago 4. 1-2 months ago 5. More than 2 months ago |
| A03 | What are the results of the swab test or PCR test? | 1. Positive 2. Negative 3. Results not yet returned 4. I don’t know 5. Unwilling to answer |
| A04 | Have you ever had a rapid antibody test or serology test with blood sampling? | 1. Yes, I have. 2. No, I haven’t 🡪 *Continue to question B01* |
| A05 | When was the rapid antibody/serology test done? | 1. Less than 1 week ago 2. 1-2 weeks ago 3. 3-4 weeks ago 4. 1-2 months ago 5. More than 2 months ago |
| A06 | What was the result of the last rapid test? | 1. Reactive 2. Non-reactive 3. Results not yet returned 4. I don’t know 5. Unwilling to answer |
| **BLOCK B** | **Symptoms Over The Last 14 Days** |  |
| B01 | In the last 14 days, have you experienced any of the following symptoms?  **(YOU MAY CHOOSE MORE THAN ONE ANSWER)** | - Loss of ability to smell or taste  - Fever  - Muscle ache  - Chills  - Cough  - Runny nose or stuffy nose (rhinorrhoea)  - Headache  - Night sweats  - Diarrhea  - Sore throat  - Difficulty breathing  - Chest pain  - Rapid heartbeat  - Phlegm (sputum)  - Dizziness  - Nausea  - Vomit  - Stomach pain  - Watery/red/swollen eyes  - Skin rash/skin redness  - Loss of appetite  - Tingling and/or numbness  - Mental confusion (delirium)  - Seizures (non congenital)  - Feeling unwell (malaise)  - Excessive fatigue  - Sudden loss of hearing  - Red eye  - No symptoms 🡪 *Continue to question C01* |
| B02 | When did the earliest symptoms appear? | - 0 day ago (started today) - 1 day ago - 2 days ago - 3 days ago - 4 days ago - 5 days ago - 6 days ago - 7 days ago - 8 days ago - 9 days ago - 10 days ago - 11 days ago - 12 days ago - 13 days ago |
| **BLOCK C** | **Illness History** |  |
| C01 | Are you currently pregnant?  **(If 2.11 = 2)** | 1. Yes 2. No |
| C02 | Do you currently have the following conditions/diseases based on a doctor's diagnosis?  **(YOU MAY CHOOSE MORE THAN ONE ANSWER)** | - Lung disease  - Stroke or brain and blood vessel diseases  - Kidney failure  - Coronary heart or other heart disease  - Diabetes mellitus  - Hypertension/high blood pressure  - Cancer  - Liver disorder  - Immune system disorder  - Acute seizure  - Other endocrine disorders  - HIV  - Others, please specify ………………  - No comorbid conditions/diseases |
| C03 | In the last 1 month, did you smoke tobacco? | 1. Yes, every day  2. Yes, not every day  3. No 🡪 *Continue to question C05* |
| C04 | In the last 1 month, how many cigarettes did you smoke on average per week? | (open-ended) |
| C05 | Did you, before the past month, smoke tobacco? | 1. Yes, I smoked every day  2. Yes, I smoked but not every day  3. Never |
| C06 | Before deciding to quit smoking, how many cigarettes did you smoke on average per week?  **(If C03 = 3 AND C05 = 1 or 2)** | (open-ended) |
| C07 | In the last 14 days, have you had contact with someone who has a fever, cough/cold, or shortness of breath? | 1. Yes, I have 2. No 🡪 *Continue to question D01* |
| C08 | If yes, please state who it is and what is your relationship with them  **(YOU MAY CHOOSE MORE THAN ONE ANSWER)** | 1. Husband/wife 2. Children 3. Parents 4. Other people living in the same house 5. Neighbors 6. Co-workers 7. Others, please specify ………………….. |
| **BLOCK D** | **Hygiene Behavior, Use of Personal Protective Equipment (PPE), and Maintaining Distance** |  |
| Hand Washing Behavior | | |
| D01 | In the last 1 month, did you wash your hands with **soap or hand sanitizer**? | 1. Yes, always 2. Yes, often 3. Yes, sometimes 4. Never (only use water) 🡪 *Continue to question D13* |
| D02-D10 | In the past month, did you wash your hands with soap or hand sanitizer: | |
| D02 | after being in public or touching objects outside the home? | 1. Yes, always 2. Yes, often 3. Yes, sometimes 4. No 5. Never went to public places |
| D03 | before eating or preparing food? | 1. Yes, always 2. Yes, often 3. Yes, sometimes 4. No |
| D04 | after using the toilet? | 1. Yes, always 2. Yes, often 3. Yes, sometimes 4. No |
| D05 | after touching animals? | 1. Yes, always 2. Yes, often 3. Yes, sometimes 4. No 5. Never touched animals |
| D06 | after touching or disposing of garbage? | 1. Yes, always 2. Yes, often 3. Yes, sometimes 4. No 5. Never disposed of garbage |
| D07 | before putting on your mask? | 1. Yes, always 2. Yes, often 3. Yes, sometimes 4. No 5. Never wore the mask 🡪 *Continue to question D09* |
| D08 | after taking off your mask? | 1. Yes, always 2. Yes, often 3. Yes, sometimes 4. No |
| D09 | after receiving goods from outside the home (courier packages, goods from neighbors, groceries, change, etc.)? | 1. Yes, always 2. Yes, often 3. Yes, sometimes 4. No 5. Never received goods from outside home |
| D10 | after shaking hands with other people? | 1. Yes, always 2. Yes, often 3. Yes, sometimes 4. No 5. Never shook hands with other people |
| D11 | In the past month, did you always carry hand sanitizer with you when you left the house? | 1. Yes, always 2. Yes, often 3. Yes, sometimes 4. No/I don’t have hand sanitizer 🡪 *Continue to question D13* |
| D12 | Can you show me the hand sanitizer that you always carry with you?  **(ENUMERATOR CONDUCTS DIRECT OBSERVATION)** | 1. Yes, respondent can present the hand sanitizer 2. Respondent cannot present the hand sanitizer |
| Mask Use Behavior | | |
| D13 | In the past month, did you wear a mask when meeting other people who are not living in the same house with you?  (example: when talking to neighbors) | 1. Yes, always 2. Yes, often 3. Yes, sometimes 4. No 🡪 *Continue to question D19* |
| D14 | In the past month, what type of mask did you wear most often when meeting other people who are not living in the same house with you? | 1. N95 mask 🡪 *Continue to question D16* 2. Surgical mask/other disposable mask 🡪 *Continue to question D16* 3. Cloth mask |
| D15 | In the past month, what type of cloth mask did you wear most often? | 1. One-ply 2. Two-ply 3. Three-ply 4. I don’t know |
| D16 | In the past month, how many times did you usually change your mask when you were outside the house? | 1. More than once a day 2. Once a day 3. Once in 2 days or more 4. Did not change in the last 1 month |
| D17 | In the past month, did you take off/pull down your mask when talking to other people who are not living in the same house with you? | 1. Yes, always 2. Yes, often 3. Yes, sometimes 4. Never |
| D18 | In the past month, did you take off/pull down your mask when meeting/gathering with other people you know or respect (other than the people living in the same house with you)? | 1. Yes, always 2. Yes, often 3. Yes, sometimes 4. Never |
| D19 | **Respondents are required to wear masks when attending the serology test site.**  **Observe how the respondent wears the mask!**  **(ENUMERATOR CONDUCTS DIRECT OBSERVATION)** | 1. The mask is worn under the nose (covering the mouth and chin) 2. The mask is pulled down to the chin/neck 3. The mask covers the nose and mouth, but does not cover the chin 4. The mask is loose, leaving the left and right sides exposed 5. The mask fits snugly on the face (not sagging) AND covers the nose, mouth and chin 6. Is not wearing a mask |
| Physical Distancing Behaviour | | |
| D20 | In the past month, did you shake hands or kiss hands when you met other people who are not living in the same house with you? | 1. Yes, always 2. Yes, often 3. Yes, sometimes 4. No |
| D21 | In the past month, did you keep a distance of at least 1 meter from other people who are not living in the same house with you? | 1. Yes, always 2. Yes, often 3. Yes, sometimes 4. No |
| D22 | In the past month, did you keep a physical distance of at least 1 meter from other people when you were wearing a mask?  **(If D13 = 1 or 2 or 3)** | 1. Yes, always 2. Yes, often 3. Yes, sometimes 4. Never |
| D23 | In the past month, did you gather with more than 2 people who are not living in the same house with you (eg. having meals together, *syukuran* (celebration, expression of gratitude), *pengajian* (recitation of the Koran), reunion, *arisan* (social gathering), etc.)? | 1. Yes, very often (more than 6 times a week) 2. Yes, often (4-6 times a week) 3. Yes, sometimes (1-3 times a week) 4. Yes, rarely (1-3 times a month) 5. Never |
| D24 | In the past month, did you travel for recreation to entertainment facilities (shopping centers, etc.), eating/drinking at restaurants/cafés, or tourist attractions (zoos, parks, car free days, etc.)? | 1. Very often (more than 6 times a week) 2. Often (4-6 times a week) 3. Sometimes (1-3 times a week) 4. Rarely (1-3 times a month) 5. Never |
| D25 | In the past month, how often did you observe acts of worship in public places of worship (mosques, churches, temples, etc.)? | 1. Very often (more than 6 times a week) 2. Often (4-6 times a week) 3. Sometimes (1-3 times a week) 4. Rarely (1-3 times a month) 5. Never |
| D26 | In the past month, did you take mass public transportation (eg. city transportation, MRT, bus, or commuter line)? | 1. Very often (more than 6 times a week) 2. Often (4-6 times a week) 3. Sometimes (1-3 times a week) 4. Rarely (1-3 times a month) 5. Never |
| D27 | In the past month, how often did you shop for basic necessities at the market or supermarket? | 1. Very often (more than 6 times a week) 2. Often (4-6 times a week) 3. Sometimes (1-3 times a week) 4. Rarely (1-3 times a month) 5. Never |
| D28 | In the past month, how often did you visit a health facility (clinic, puskesmas, hospital, doctor’s/midwife’s private practice)? | 1. Very often (more than 6 times a week) 2. Often (4-6 times a week) 3. Sometimes (1-3 times a week) 4. Rarely (1-3 times a month) 5. Never |
