# Supplementary material for: Seroprevalence of antibodies against SARS-Cov-2 in the high impacted sub-district in Jakarta, Indonesia
Source: PLoS One. 2021 Dec 23;16(12):e0261931. doi: 10.1371/journal.pone.0261931 (PMC8699601; doi:10.1371/journal.pone.0261931)
Supplement: S1 File — (DOCX) [file pone.0261931.s002.docx]

**S1 File. Research summary, challenges, and prospects**

| **Summary** | **Challenges** | **Prospects for further studies** |
| --- | --- | --- |
| The purpose of the present study was to observe the actual prevalence and infection fatality rate (IFR) of COVID-19 in Tanjung Priok subdistrict, Jakarta. We found that the estimated actual transmission in the area is far higher than the reported figure by the time of data collection. Now that the pandemic remains unabated, the result of this study provides a clearer picture on the stage of the COVID-19 transmission in Tanjung Priok by February 2021 and improvement on the surveillance system in the area is required to have a better understanding and response to the pandemic. | 1. The test kit utilized does not provide information on the type of anti-SARS-CoV-2 antibodies present (IgG and IgM) 2. Several conditions in the field affect the longer data collection period which might suffer pandemic dynamics due to potentially increased mobility during an end-of-year holiday | 1. Using advanced antibody test kits will enable further researchers to examine different types of antibodies against SARS-CoV-2, as well as the infection duration. 2. Taking the period of data collection into account will provide a more accurate result of seroprevalence 3. Larger seroprevalence studies in wider areas in Indonesia may help in understanding the actual transmission in the population |
