# Supplementary material for: Seroprevalence of antibodies against SARS-Cov-2 in the high impacted sub-district in Jakarta, Indonesia
Source: PLoS One. 2021 Dec 23;16(12):e0261931. doi: 10.1371/journal.pone.0261931 (PMC8699601; doi:10.1371/journal.pone.0261931)
Supplement: Attachment — Submitted filename: Response to reviewers.docx [file pone.0261931.s003.docx]

December 6th, 2021

**Rebuttal Letter to manuscript PONE-D-21-30389**

**“Seroprevalence of Antibodies against SARS-CoV-2 in the High Impacted Sub-district in Jakarta, Indonesia”**

Olivia Herlinda, Adrianna Bella, Gita Kusnadi, Dimitri Swasthika Nurshadrina, Mochamad Thoriq Akbar, Sofwatun Nida, Ngabila Salama, Iwan Ariawan, Diah Saminarsih

Dear Editor Dr. Sanjay Kumar Singh Patel, and all reviewers

We thank the editor and all reviewers for taking their valuable time to give generous comments and suggestions on the manuscript (MS), which gave us the opportunity to improve the paper. Please find our attempts to answer all the comments and suggestions raised by the Editor and the reviewers below. Comments are shown in bold and italic font, followed by our answer/comment in normal font. The major corrections/changes in the manuscript are displayed in red font. In the revised manuscript, we hope to address all the issues raised.

***Editor’s comments:***

***Dear Dr. Bella,***

***Journal Requirements:***

***When submitting your revision, we need you to address these additional requirements.***

***1. Please ensure that your manuscript meets PLOS ONE's style requirements, including those for file naming. The PLOS ONE style templates can be found at***

[***https://journals.plos.org/plosone/s/file?id=wjVg/PLOSOne_formatting_sample_main_body.pdf***](https://journals.plos.org/plosone/s/file?id=wjVg/PLOSOne_formatting_sample_main_body.pdf) ***and***

[***https://journals.plos.org/plosone/s/file?id=ba62/PLOSOne_formatting_sample_title_authors_affiliations.pdf***](https://journals.plos.org/plosone/s/file?id=ba62/PLOSOne_formatting_sample_title_authors_affiliations.pdf)

***2. If your study included minors, state whether you obtained consent from parents or guardians. If the need for consent was waived by the ethics committee, please include this information.***

***3. Please include additional information regarding the survey or questionnaire used in the study and ensure that you have provided sufficient details that others could replicate the analyses. For instance, if you developed a questionnaire as part of this study and it is not under a copyright more restrictive than CC-BY, please include a copy, in both the original language and English, as Supporting Information.***

***4. Thank you for stating the following financial disclosure:***

***“This study is fully funded by the Center for Indonesia's Strategic Development Initiatives (CISDI), where most authors work as researchers, as one of its research projects. We disclose that the institution is an independent think tank organization which does not have any conflict of interest with any potential external bodies.”***

***Please state what role the funders took in the study.  If the funders had no role, please state: "The funders had no role in study design, data collection and analysis, decision to publish, or preparation of the manuscript."***

***If this statement is not correct you must amend it as needed.***

***Please include this amended Role of Funder statement in your cover letter; we will change the online submission form on your behalf.***

***5. Please review your reference list to ensure that it is complete and correct. If you have cited papers that have been retracted, please include the rationale for doing so in the manuscript text, or remove these references and replace them with relevant current references. Any changes to the reference list should be mentioned in the rebuttal letter that accompanies your revised manuscript. If you need to cite a retracted article, indicate the article’s retracted status in the References list and also include a citation and full reference for the retraction notice.***

Dear Editor,

We thank you for the comments given to our MS which improved the quality of our paper significantly. On your suggestion to upload our lab protocol, unfortunately we did not do any lab procedures so there is no lab protocol available.

Being specific on the other points you raised:

#1. We revised the manuscript style and file naming accordingly.

#2. We included minors aged 15-17 years old and had obtained the consent through their guardians.

#3. We have uploaded the questionnaire (in Indonesian and English) we used in this study into the system as the supplementary information (S1_Appendix). We have also added a new heading titled “Supporting information” in the manuscript.

#4. We have added the following statement "This study is fully funded by the Center for Indonesia's Strategic Development Initiatives (CISDI). We disclose that the institution is an independent non-governmental organization which does not have any conflict of interest with any potential external bodies. The funder had no role in study design, data collection, and data analysis."

#5. We have rechecked all the references and certain everything is in correct order and complete.

***Reviewers' comments:

Reviewer's Responses to Questions***

***Comments to the Author

1. Is the manuscript technically sound, and do the data support the conclusions?

The manuscript must describe a technically sound piece of scientific research with data that supports the conclusions. Experiments must have been conducted rigorously, with appropriate controls, replication, and sample sizes. The conclusions must be drawn appropriately based on the data presented.***

***Reviewer #1: Yes***

***Reviewer #2: Yes***

***2. Has the statistical analysis been performed appropriately and rigorously?***

***Reviewer #1: Yes***

***Reviewer #2: Yes***

***3. Have the authors made all data underlying the findings in their manuscript fully available?

The*** [***PLOS Data policy***](http://www.plosone.org/static/policies.action#sharing) ***requires authors to make all data underlying the findings described in their manuscript fully available without restriction, with rare exception (please refer to the Data Availability Statement in the manuscript PDF file). The data should be provided as part of the manuscript or its supporting information, or deposited to a public repository. For example, in addition to summary statistics, the data points behind means, medians and variance measures should be available. If there are restrictions on publicly sharing data—e.g. participant privacy or use of data from a third party—those must be specified.***

***Reviewer #1: No***

***Reviewer #2: Yes***

Dear Editors,

We have added the following statement in the data availability:

“The data is owned by the Center for Indonesia's Strategic Development Initiatives (CISDI) and can be made available for scientific purposes upon request. A data sharing agreement will be needed to ensure privacy and data handling. A request for deidentified data and a data dictionary can be requested to the corresponding author ([adrianna.bella@cisdi.org](mailto:adrianna.bella@cisdi.org)) or [secretariat@cisdi.org](mailto:secretariat@cisdi.org).”

***4. Is the manuscript presented in an intelligible fashion and written in standard English?

PLOS ONE does not copyedit accepted manuscripts, so the language in submitted articles must be clear, correct, and unambiguous. Any typographical or grammatical errors should be corrected at revision, so please note any specific errors here.***

***Reviewer #1: Yes***

***Reviewer #2: Yes***

***5. Review Comments to the Author

Please use the space provided to explain your answers to the questions above. You may also include additional comments for the author, including concerns about dual publication, research ethics, or publication ethics. (Please upload your review as an attachment if it exceeds 20,000 characters)***

***Reviewer #1: This investigation uses serology testing to detect the antibodies against SARS-CoV-2 in the population of Tanjung Priok in Jakarta, Indonesia to estimate the seroprevalence and infection fatality rate (IFR) in the community. The results show that the prevalence of antibody against SARS-CoV-2 was 28.52%. The estimated IFR was 0.08%. The big difference between the estimated COVID-19 seroprevalence and the reported cumulative cases in Tanjung Priok shows significant under-reporting of COVID-19 cases in this community. As this study presented some interesting information on the estimated percentage of people infected by SARS-CoV-2 in Tanjung Priok sub-district of Jakarta, it may be published yet not in priority. This is because, in the current stage of the pandemic, after several waves of SARS-COV-2 virus breakout worldwide, understanding the actual prevalence of COVID-19 transmission in the community might not be strategically important in fighting the COVID-19 pandemic when most of the COVID-19 cases are asymptomatic or mild. Contrary to the authors suggestion of putting more efforts to amplify epidemiological surveillance by the provincial and local governments, more efforts and resources should be reserved for the care and treatment of the severe cases of COVID-19 patients to save lives.***

Dear reviewer #1, we thank you for the general appreciation of our work, and specific comments given that help to improve our manuscript. We believe it is important to understand the scale of this pandemic through seroprevalence survey as part of evaluation process of government policies and interventions. Ultimately, whether the testing and tracing capacity have been sufficient, which then may provide estimate how much more it should be improved. As well, though it is still in the central of debate, seroprevalence can be the basis for the government to pursue how much more vaccination rates should be moved forward, as well as in which demographics. Related to your further comment, we agree that by knowing the scale of the infection, the government can calculate and predict how much more care and treatment should be prepared for the coming wave, which we have incorporated in line 327-329 in the file named “Manuscript”.

***Reviewer #2: In the current research article entitled " Seroprevalence of Antibodies against 1 SARS-Cov-2 in the High Impacted Sub-district in Jakarta, Indonesia", by Herlinda et al., have studied/surveyed estimate the seroprevalence and infection fatality rate (IFR) in Tanjung Priok, the hardest-hit sub-district by the COVID-19 in Jakarta, Indonesia. Authors conducted venous blood sampling to 3,196 individuals in Tanjung Priok between Nov 23, 2020, and Feb 19, 2021. They found that, under-reporting is an issue between the estimated COVID-19 seroprevalence and the reported cumulative cases in Tanjung Priok. This article addresses a research topic of great interest, which is under intense investigation in the past 2 years and the manuscript is generally well-written. However, this reviewer has certain suggestions that would help produce a more comprehensive overview of the topic:

Suggestions:

1. The authors may additionally provide one Figure as summary, challenges, or prospect of the present study.
2. The authors should cross-check all abbreviations in the manuscript. Initially, define in full name followed by abbreviation.
3. The English of manuscript can be polished (minor).***

Dear Reviewer #2,

We thank you for your generous comments. To address some of your specific points:

#1. We have provided the Figure as a supplementary file (S2_File). We have also added a new heading titled “Supporting information” in the manuscript.

#2. We have rechecked it again and make sure that 1) all abbreviations are mentioned when the spelled-out versions first appear in the manuscript and 2) all abbreviations are uniform throughout the manuscript, thank you for the kind reminder.

#3. Thank you, we have rechecked the accuracy of language that we used in the manuscript. Changes were tracked in the file “Revised Article with Changes Highlighted”.
